# Supplementary material for: Anatomically aware simulation of patient-specific glioblastoma xenografts
Source: PLoS Comput Biol. 2026 Jan 20;22(1):e1013831. doi: 10.1371/journal.pcbi.1013831 (PMC12851450; doi:10.1371/journal.pcbi.1013831)
Supplement: Attachment — Submitted filename: Response_to_reviewers.pdf [file pcbi.1013831.s002.pdf]

Reviewer 1

Response: We would first like to thank the reviewer for their valuable and high-quality feedback. Below are copies of the comments, along with our responses.

General summary: In this article the authors present a method to estimate tumor growth model parameters from histological sections from patient-derived xenografts. Using four different PDX models with 2 replicates each, the authors collected tissue sections which were subsequently binarized for comparison against the output of an agent based model. The agent base model described tumor growth and invasion which was sensitive to white matter fiber tracts (DTI collected in a different animal) and tissue-cleared maps of vasculature (Also collected in different animals). The authors then applied approximate bayesian computation (ABC) requiring sampling parameter ranges to yield 5000 simulations per animal, the top 1% (50) were selected using a geometry based measure. In general, this is a very interesting paper that presents a novel contribution to Computational Biology/Mathematical Oncology demonstrating an approach for comparing agent based model results to experimental data. I do have several concerns that should be addressed before further consideration.

Comment 1: Comparison to existing literature and selection of method: In the introduction you provide an overview of existing PDE (proliferation-invasion) and ABM based models. What is the main motivation for using ABM methods over PDE methods for these type of problems? This is covered in the discussion, but some of this topic should be covered in the introduction to help support the modeling choices made in this manuscript. Some PDE-based methods to consider discussing that include vasculature-coupled growth and/or white matter coupled growth:

(1) Human application: Hawkins-Daarud A, Rockne RC, Anderson AR, Swanson KR. Modeling Tumor-Associated Edema in Gliomas during Anti-Angiogenic Therapy and Its Impact on Imageable Tumor. Front Oncol. 2013 Apr 4;3:66. doi: 10.3389/fonc.2013.00066. PMID: 23577324; PMCID: PMC3616256.

(2) Mice/rats: Hormuth, D.A., Jarrett, A.M., Feng, X. et al. Calibrating a Predictive Model of Tumor Growth and Angiogenesis with Quantitative MRI. Ann Biomed Eng 47, 1539–1551 (2019). https://doi.org/10.1007/s10439-019-02262-9

(3) Mice/rats: Rutter, E.M., Stepien, T.L., Anderies, B.J. et al. Mathematical Analysis of Glioma Growth in a Murine Model. Sci Rep 7, 2508 (2017). https://doi.org/10.1038/s41598-017-02462-0

Response: We included a motivation of using an ABM in the introduction on page 3. We also

included the suggested references 1 and 2, but did not include 3 as it appears to be of less relevance for the current work.

Comment 2: Third paragraph introduction: I think there is a word or phrase missing here. “This paper seeks to align two distinct approaches to brain tumor invasion”. Is it “two distinct approaches to model/study brain tumor invasion”?

Response: You are correct. This has now been fixed.

Comment 3: Missing figure label and or caption? At the top of page 6, there are two panels showing the 3D maps of white matter and the vasculature. These should be given a figure number and referenced in the text.

Response: Thanks for pointing this out. We have added an in-text reference on page 5.

Comment 4: Figure 3 is missing a figure caption.

Response: Thanks for pointing this out. We have now added a caption.

Comment 5: Section 2.2, third paragraph. On the first read it is not clear why 50 accepted parameters were chosen. I would provide more background in this section to say how many total simulations/samples were collected and what percent of those you saved. (You might add this in your discussion of ABC in section 2.1.

Response: We now clearly state this at the end of the fourth paragraph in section 2.1.

Comment 6: Section 2.3, Some of the comparisons between replicates in this section are vague or contradictory to the results shown in Table 1. For example,for U3230MG you state that the parameters agree well except for w_{bv}, but the median or mean value of p_m is nearly 2 times different between the replicates. Similar comments for U3062MG.

Response: We have now revised the descriptions, in accordance with the table and your comments.

Comment 7: Section 2.3, I would consider defining delta here as the acceptance threshold to improve the clarity of this section.

Response: We added this in parentheses to remind the reader that this is the acceptance threshold.

Comment 8: For figure 4, I would consider adding the error or the value of the Jaccard score to provide reference quantifications for the best fits.

Response: This is a good suggestion. We have now added the Jaccard index and the geometric distance/error in the subfigure captions of Figure 4.

Comment 9: Section 2.5. I am not entirely convinced that this section is needed for this manuscript. Is the proposed in silico drug mimicking a clinical available option? If not, is there a reason why standard of care treatment (e.g., fractionated radiotherapy and/or temozolomide) options were not modeled on with this framework

Response: The purpose of the section is to demonstrate (i) that it is possible to simulate treatment options based on reduced migration/proliferation, and (ii) to show that the responses to such hypothetical drugs/combinations is heterogeneous, and not the same for all tumors. As the reviewer proposes, it would be very interesting to do a large systematic comparison between both standard of care treatment (which could include both drugs, radiation as well as surgical intervention) and hypothetical drug combinations, this lies outside the scope of the current work.

Comment 10: Section 3.1, "data" should be changed to "Data".

Response: Done!

Comment 11: Section 3.2. Second paragraph. What are the units of the radius of 8? (8 microns, lattice/grid positions?). How closely does this radius of 8 correspond to the volume of injected cells. Does the volume/radius of injected cells in the initial condition influence the estimated model parameters? Have you considered using an ellipsoid to model the initial condition (assuming cells are distributed along the injection track rather than at a precise location?

Response: We now describe that it is 8 grid points we mean. We did not investigate the influence of ellipsoid or other initial configurations, but this is a valid point. There is reason to believe that, in certain instances, cells may indeed end up in the injection track. However this is not something we investigated in this study, but would be interesting at a later stage. This is mentioned in the discussion.

Comment 12: Section 3.3, "We choose a carrying capacity of K = 3". How much does the selection of K influence the estimated parameters? Would a higher K (K = 5 or 10) or a lower K (K = 1 or 2) skew the ratio of the probability to proliferate and probability to migrate?

Response: This is a very interesting but difficult question. In short, we do not know exactly the impact of varying the carrying capacity on the estimated parameters. What we do know is that it depends on many factors, such as choice of summary statistic as well as the model itself, as well as the initial conditions. The complete exploration of this topic is outside the scope of this paper. However, based on experience working with this framework, we believe that the effects are limited for two reasons. The first is that we effectively normalize (by binarizing the images) before

comparing simulation to experiment. Secondly, the geometric measure does not take into account e.g. cell count (which would be heavity influenced by choice of K.). We believe that the geometrical properties such as number of connected components, area and eccentricity are less influenced by the choice of K, especially when applied to a binarized image that does not take actual cell numbers into account.

Comment 13: Discussion, Is only one tissue section available from the experimental data? If so, is a 3D simulation truly needed to estimate these parameters, and if not could the number of cells in the 2D simulation be increased?

Response: This is an interesting point. For almost all mice there are five sections available. However, many of them are damaged. In order to achieve a decent number of samples (in our case 4 + 4) we would need to use a varying number of slices. This would heavily influence the results and would make it impossible to compare model fit between animals. In addition, for each slice we use, additional error are introduced (due to picking which section in the simulation brain it corresponds to). Regarding 2D vs. 3D modelling, it would have been a tremendous improvement in computational requirements (less demanding) if we went with 2D. However, we believe that a 2D section does not fully capture the intricate structures of the brain. Whatever the cells would perceive in a given 2D section is not representative of the real 3D anatomy.

Reviewer 2

This manuscript presents a promising computational approach that explicitly incorporates both white matter and blood vasculature to predict glioblastoma xenograft growth. The overall structure and summary of the work are well articulated. However, the manuscript would benefit from clarification and elaboration in several key areas.

Response: Thanks a lot for providing this valuable feedback on our paper. Please find our responses below.

Major Comments:

1) In the Results section, there is ambiguity in the use of the terms metric, method, and algorithm within the ABC framework. The manuscript should clarify that the same ABC rejection algorithm is used throughout, with a fixed tolerance (e.g., 1% acceptance rate). It is unclear what the authors mean by "regression adjustment"—does this refer to the use of the Epanechnikov kernel, or to a change in the summary statistics used to compare geometric metrics? Please revise

the following paragraph accordingly, and ensure consistent terminology throughout the manuscript: "Given such 'ground truth' data, we evaluated three versions of the ABC algorithm. First, in the simplest version, we matched simulations to experiments using the Jaccard index (the fraction of tumor-containing pixels that overlapped between experiment and simulation). We further proposed a geometric similarity measure with regression adjustment (as explained in Section 3.3), and a 'direct' method without regression adjustment." In addition, the explanation of regression adjustment in Section 3.3 is not sufficiently clear. More detail, possibly in the Supplementary Information (SI), would be helpful.

Response: Thanks for pointing out the ambiguity. We have now clarified what we mean. We specify that it is a rejection-ABC, but with 2 different summary statistics, and in the third case we also use postprocessing in the form of regression adjustment. This is explained in detail with references in Section 3.3. We are uncertain what the reviewer means by the description not being sufficiently clear. We have a reference to a textbook where the details of regression adjustment are described, and have also detailed the transformations we use (logit) and the kernel and bandwidth used.

2) In Section 2.4, it is interesting that the full model demonstrated greater plausibility than the white-matter-only model for tumors 4, 5, and 7. Since these are the largest tumors, could this be due to their increased dependence on vasculature? A discussion of the biological rationale behind this finding would strengthen the manuscript.

Response: This is indeed a very interesting question, however it is difficult to answer with certainty. As mentioned, tumors 4 and 5 are among the largest and has the highest proportion of accepted parameters from the full model (including vascular structures). However, tumors 3 and 6 are about the same size but have a lower proportion of parameters from the full model. Furthermore, the model does not assume that proliferation increases in the vicinity of vasculature at this point. Although this would be a great extension, it would also make the model significantly more complex, at least if e.g. nutrients/oxygen concentration was modelled explicitly.

3) If the time scale is adjusted for each experiment, how can the model be used to make predictions in scenarios where no prior data are available? Since the model uses a real-time unit, one could potentially estimate the growth pattern from other data sources (e.g., RNA-seq). Additionally, is the spatial unit also adjusted for each experiment? Please clarify.

Response: As the reviewer pointed out, without any prior information at all, it is not possible to make predictions. However, it is a very good and interesting idea to try to utilize e.g. RNA-seq data. The connection between migration/proliferation/morphology and e.g. RNA-seq data is a problem of utmost importance, and this is something we would like to explore. The challenge lies in the number of mice/sample size required to obtain statistically meaningful results. However,

the model has utility since it can quantify the tumor properties (thorugh the four parameters) and can potentially be used to make predictions forward in time in case the tumor size/shape has been observed once.

4) The manuscript lacks information regarding model uncertainty. Given that this is a stochastic model, how many replicates per parameter set are required to produce an accurate summary statistic of the model output?

Response: Given that it is a stochastic model this is a valid point. However, we have not made quantitative investigations into this issue. Based on experience and observation, there is not much variation between simulations with fixed parameters. In addition, the parameter estimation procedure captures, among other sources of variability, the stochasticity of the model, i.e. if there had been a large variability between runs with the same or similar parameters, this would cause large errors in the fitting procedure.

Minor Comments:

1) In the last paragraph of Section 2.2, please describe the meaning of "formula 4 and 5" to improve readability and flow.

Response: Formula 4 is currently described in the text as "the relative error between the true parameters ... and the estimated parameters". Formula 5 is then described as "The overall error for a specific method is then the average error over the 50 accepted parameters". It is unclear how we can clarify this further.

2) Figures 2 and 3 lack captions and are not clearly referenced in the main text.

Response: Thanks for pointing this out. It has now been fixed.

3) In Section 2.5, the authors state: "For tumors 1–4 and 6, it appears that the cell count is lowest when the tumor is treated with only anti-migration or only anti-proliferation drugs, with a maximum somewhere between the two extreme cases." Does this observation not apply to tumor 5? Please clarify.

Response: We realize that our phrasing caused confusion. We have rewritten this description. As you pointed out, the observation for cell count indeed holds true for tumor 5 as well. We see how our sentence may have been confusing. Indeed for tumors 1-4 and 6, the cell count has a maximum at intermediate doses, and approximately equally low counts for both pure anti migratory and pure anti proliferatory treatments. As you pointed out, in terms of cell count this

4) In SI Section 5.2.4, the comparison steps need more detail: Step 1: What criterion was used

for decision-making? Was it based on error? Step 2: What technique was used in the binarization step? Step 4: Please describe the “closest point” method in one sentence to give readers a quick understanding. Step 5: Provide more detail about how the transformation was applied.

Response: Thanks for this feedback. We have now revised and updated this section to more clearly describe the preprocessing steps. We also modified the images (although they illustrate the process in a similar way) to more closely match the description of the steps. Notice that we also replaced the three small subfigures on figure 1D to match these images as well. We also updated a small part of Figure 1 to use the same images, to more closely resemble the actual processing steps.
