# Supplementary material for: Anatomically aware simulation of patient-specific glioblastoma xenografts
Source: PLoS Comput Biol. 2026 Jan 20;22(1):e1013831. doi: 10.1371/journal.pcbi.1013831 (PMC12851450; doi:10.1371/journal.pcbi.1013831)
Supplement: Attachment — Submitted filename: Reponse letter_v2.pdf [file pcbi.1013831.s003.pdf]

Reviewer 2

The following points were addressed satisfactorily in the response but should still be integrated into the revised manuscript:

Response: We thank the reviewer for these suggestion and we have now included large parts of our responses into the main manuscript.

Q2) The authors provide a satisfactory conceptual response, but this point should be explicitly mentioned in the Discussion section as a potential model extension and limitation, particularly regarding proliferation dependency on vascular proximity and nutrient/oxygen availability.

Response: This has been added to the Discussion section, where we discuss future extensions.

Q3) The response is satisfactory and well reasoned, but the discussion should be expanded in the manuscript to reflect these limitations and potential extensions (e.g., need for prior data, possible integration of RNA-seq).

Response: This is also now added to the last paragraph in the discussion section.

Q4) The justification is sound, but the manuscript should explicitly mention that one replicate per parameter set was used because of observed low variance, and that parameter estimation accounted for this low stochastic variability.

Response: This is explicitly mentioned, and our justification included on page 18 in the manuscript, where we discuss the ABC protocol.

Minor comment:

Q3) The authors' response indicates the description has been rewritten, but I cannot find this change in the revised manuscript. The clarification therefore appears incomplete. Please update the manuscript to include the full corrected explanation and indicate where this change was made.

Response: We have rewritten this paragraph to clarify our observations, and at the same time corrected the fact that also tumor 5 exhibits the same qualitative form as tumors 1-4 and 6. The changes are marked in the revised manuscript. The canges can be found in the top paragraph of p. 13.

In addition to the above we found a small number of grammar mistakes and inconsistencies with wording. The changes are

1. tumours → tumor p. 22.

2. position → positions p. 22.

3. agent based → agent-based (multiple places) p. 23.

4. correlates → correlate p. 22.

These changes have not been marked in blue.
